# Effect of Different Method of Drying of Five Varieties Grapes (*Vitis vinifera* L.) on the Bunch Stem on Physicochemical, Microbiological, and Sensory Quality

**DOI:** 10.3390/foods9091183

**Published:** 2020-08-26

**Authors:** Radka Langová, Miroslav Jůzl, Olga Cwiková, Ivica Kos

**Affiliations:** 1 Department of Food Technology, Faculty of AgriSciences, Mendel University in Brno, 61300 Brno, Czech Republic; radka.langova@mendelu.cz (R.L.); olga.cwikova@mendelu.cz (O.C.); 2 Department of Animal Science and Technology, Faculty of Agriculture, University of Zagreb, 10000 Zagreb, Croatia; ikos@agr.hr

**Keywords:** grapes, drying, lyophilisation, spectrophotometry, sensory analysis, microbiology, colour

## Abstract

The influence of the drying technique on certain quality attributes of dried grapes was analysed. Five varieties of grape were used in this study (Bezsemenné, Perlette, Vrboska, Beauty seedless, and Jakubské). All the varieties were dried using four methods: drying at 40 °C, combined drying (70, 65, and 60 °C), drying at 70 °C, and lyophilisation. The quality attributes such as total soluble solids, water activity, content of vitamin C, the colour parameters (L*, a*, b*), and microbiological (colony forming units, moulds, yeasts) and sensory (smoothness, gloss, colour, odour, chewability, juiciness, flavour, and overall impression) analysis were determined, depending on the method of drying. Lyophilised grapes from the Jakubské variety had the highest vitamin C content. They contained 0.58 mg of vitamin C per 100 g of dried grapes. However, no significant differences between varieties in vitamin C content were found. This confirmed concerns about the negative impact on the nutritional quality of dried grapes with increasing temperature. The lowest total count of microorganisms (CFU), moulds, and yeasts were determined after using highest drying temperature (70 °C). Higher CFU values were determined in lyophilised grapes, and then followed by grapes dried at 40 °C. Lyophilised grapes and grapes dried at 40 °C achieved the best sensory rating in general. The final product was intended to have a unique appearance while maintaining the evaluated quality parameters.

## 1. Introduction

Consumption of grapes and raisins dates to prehistoric times, which is proven by rock paintings from the Mediterranean region [1]. It is assumed that the first raisins were produced in the Near East [2]. The purpose of grape drying was to extend its storage life and, with regard to their high sugar content, to provide a rich source of energy for workers. Raisins were also valued because they were easy to transport [1,2].

Grapes and products made from grapes are among the most important horticultural products in the world. They are grown on all the inhabited continents on Earth [3,4,5,6]. Approximately 75 million tons of grapes are produced every year. Nearly 50% of grapes are processed into wine, must, and juice; one third is consumed as fresh fruit; and the rest are dried and processed into other products [5]. World production of dried grapes reached 1.2 million tons in the 2018/2019 season [7], and it continues to increase [2,5].

Drying is one of the oldest and simplest methods of food conservation. It is a gentle method of preservation by which beneficial aromatic and content properties of the food are retained. After drying, food gains a longer storage life, becomes lighter, and its volume decreases [8,9,10]. Grapes are susceptible to drying and are traditionally dried in the sun for 8 to 10 days. This method of drying is cheap, but there is a risk of the grapes being infested by insects or contaminated. An alternative and advanced method is drying with hot air, while other techniques include dehydration using microwave radiation, vacuum drying, or infrared drying [2,8,10,11]. The reduction of water activity in dried grapes is of greatest importance, because the products become more stable and with storage under appropriate conditions the optimum microbiological, nutritional, and sensory properties of the finished product are achieved [8,12]. The quality of dried grapes can be described through many properties, but microbiological safety should be assured considering that dried grapes could be contaminated by filamentous micromycetes, which can produce mycotoxins [13].

Dried grapes have valuable nutritional properties because of high mineral, vitamin, and other bioactive substance content. Therefore, current research indicates that grape consumption has many health benefits such as the reduction of the risk of cancer, type 2 diabetes, stroke, obesity, cardiovascular diseases, and other chronic diseases [3,10,14,15]. The health benefit of vitamin C is clear; nevertheless, the effects of some phenolic polymers found in raisins, grapes, and wine in humans are still questionable [16]. Grapes have a high carbohydrate content and relatively high energy value but a low glycaemic index, which makes grapes suitable for special diets [5].

Dried grapes are excellent sources of health-promoting bioactive compounds, but their quality can be negatively affected during drying. The aim of this study was to compare several drying techniques and decide, whether there are differences between the drying techniques in selected nutritional (vitamin C content), microbial, and sensory quality parameters for five varieties typical from the region.

## 2. Materials and Methods

### 2.1. Materials

Five varieties of grapes were used in this research: Jakubské (JA), Beauty seedless (BT), Vrboska (VR), Bezsemenné (BS), and Perlette (PE). The varieties are described in Table 1 according to Vitis International Variety Catalogue (VIVC). Samples of grapes were obtained from the Ampelos grape cultivation station, which is located in Vrbovec near Znojmo (48°48′3.47″ N, 16°4′54.27″ E, Czech Republic) in August 2018, at the time of their full maturity.

For the experiments, 7 kg of ripened grapes on the bunch stem from each variety was carefully taken and randomly divided equally among each method of drying.

### 2.2. Drying Methods

Before drying, samples were rinsed under running tap water, and the bunch stems were not separated from the berries, so the entire bunch of grapes was dried. The grape bunches were dried using 4 methods: three with air at 40 °C, at 70 °C and using a combined method (70, 65, and 60 °C) in an SO 1070 fruit drier (Concept, Choceň, Czech Republic), and one method using lyophilisation at a temperature of −42 °C and a pressure of 0.22 mbar using an Alpha 1–2 LD Plus lyophilising machine (Christ, Osterode am Harz, Germany).

The samples were dried until they reached the water activity value of a_w_ = 0.6. Water activity was determined using an electrolytic sensor on the LabSwift-aw device (Novasina AG, Lachen, Switzerland) in triplicates and with a sensor stabilisation time of 6 min. The preparation of dried samples included cutting to pieces of diameter about 4 mm.

### 2.3. Determination of pH

pH was determined on fresh grapes in triplicates at a temperature of 25 °C using a Portavo 907 MULTI pH meter (Knick, Berlin, Germany) equipped with a puncture probe.

### 2.4. Determination of Total Soluble Solids Content

Total soluble solids content (TSS) was measured in the manually pressed juice from the fresh grapes using a pocket optical refractometer (Krüss, Hamburg, Germany), with the procedure repeated three times at a temperature of 25 °C.

### 2.5. Determination of Vitamin C

Vitamin C content was determined on 10 g of crushed, homogenised, and filtered samples of fresh and dried grapes in duplicates using a solution of oxalic acid. Vitamin C was determined using a 1260 Infinity II liquid chromatograph (Agilent Technologies, Santa Clara, CA, USA) on a Luna Omega Polar C18 column, 5 µg, 250 × 4.6 mm (Phenomenex, Torrance, CA, USA). Oxalic acid (0.005 M) was used as a mobile phase and the flow-through rate of the mobile phase was 0.8 mL per minute. Ten microlitres of the sample was injected. The concentration of vitamin C was determined using a DAD (Diode-Array Detection) detector at a wavelength of 254 nm using a calibration curve. The calibration curve points were 0.1, 0.2, 0.5, 1, and 10 µg/mL. The limit of detection was 0.01 µg/mL and the limit of quantification was 0.02 µg/mL. The measurements were taken at a temperature of 25 °C.

### 2.6. Determination of Instrumental Colour

Instrumental colour measurement was determined on fresh and dried raisins. Colour was measured using a CM 3500d spectrophotometer (Konica Minolta, Tokyo, Japan), which was connected to a computer with CMs-100w SpectraMagic NX software (Konica Minolta, Tokyo, Japan). Colour was measured by using the CIE L*a*b* tristimulus colour space at two different sides of the grape berry. Grape colour measurements were performed in triplicates on 10 berries of grapes, on a slot with a measurement area of 8 mm, specular component excluded (SCE), and an observer with an angle of 8°, CIE standard illuminant D65. Results were expressed as L*, a*, and b* values (CIELab coordinates). The L* represents lightness (100 for white and 0 black), chromatic coordinate a* represents the colour space between red (+a*) and green (−a*), chromatic coordinate b* represents the space between yellow (+b*) and blue (−b*). The total colour change or difference (CIE ΔE*_ab_ in the following formula) from fresh berries to dried raisins was computed using the equation, where parameters with subscript L*_0_, a*_0,_ b*_0_ are values for the fresh grapes and L*_1_, a*_1_, and b*_1_ are values of the dried raisins, respectively. Overall total colour change ΔE*_ab_ is an important, widely accepted method of evaluating the significance of colour difference [17].

Formula:

$$∆E{*}_{\mathrm{ab}}=\sqrt{{\left(L{*}_{1}-L{*}_{0}\right)}^{2}+{\left(a{*}_{1}-a{*}_{0}\right)}^{2}+{\left(b{*}_{1}-b{*}_{0}\right)}^{2}}$$

### 2.7. Microbiological Analysis

The presence of moulds, yeasts, and the total count of microorganisms was determined on fresh berries and dried raisins in duplicates. For that purpose, 10 g of homogenised sample was taken and inserted into a homogenisation bag, and 90 mL of physiological solution was added. The samples were then homogenised in a Stomacher. Moulds and yeasts were determined according to ISO 21527-2 [18], the total number of microorganisms was determined according to ISO 4833-1 [19] using a decimal dilution series. DRBC (dichloran rose-bengal chloramphenicol agar) growth medium was used to determine and selectively analyse mould and yeast count, and PCA (plate count agar) was used for the determination of the total number of microorganisms.

### 2.8. Sensory Analysis

Sensory analysis was performed in a specialised sensory laboratory by 10 assessors aged 25 to 50 years. Assessors belonged to the staff of the Department of Food Technology at Mendel University and had previous experience in the sensory analysis of food with more than 25 sensory analyses per year. Before sensory analysis, they performed a training period in general and specific aspects on the sensory analysis of grapes and raisins. Each assessor was given 20 samples of dried raisins from every drying method and variety. Samples were served on white ceramic plates and sample order was based on a random, completely balanced design. Samples were evaluated on a graphic line scale of 100 mm with the anchoring points located at both ends. The anchored extremes for all descriptors were from low to high level of descriptor’s intensity (or hedonic). The following descriptors were evaluated: smoothness, gloss (as surface properties; intensity), colour, aroma, chewability, juiciness (all intensity), taste (hedonic: pleasantness of taste) and overall impression (hedonic). Sensory analysis was performed in two sessions.

### 2.9. Statistical Assessment

Single-factor ANOVA with standard deviation, weighted average, and Tukey’s HSD test were used for statistical evaluation using STATISTICA 12 software (StatSoft, Prague, Czech Republic). Principal component analysis (PCA) on correlation matrices with the Varimax rotation was used to find the relationships between vitamin C content, sensory descriptors, instrumental colour attributes, and water activity among raisins of different varieties and drying treatments [20].

## 3. Results and Discussion

### 3.1. pH and Total Soluble Solids Content

The Perlette variety had the highest pH (3.42), and the Bezsemenné variety had the lowest pH (3.14). It was found that the grapes of Perlette, Vrboska, and Beauty seedless varieties had significantly higher (*p* < 0.05) pH values than the grapes of Bezsemenné and Jakubské varieties. Grapes of the Vrboska variety had significantly higher TSS content than those of the Perlette variety, which had the lowest TSS content (Table 2). It is generally known in practice that grapes with a high TSS should be used for the production of high-quality dried raisins.

### 3.2. Vitamin C Content

**Figure 1:**
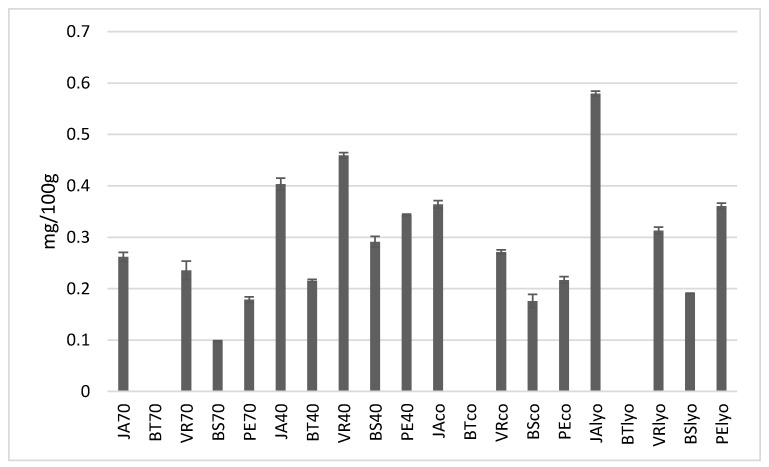
Concentration of vitamin C in individual varieties depending on drying method (70 °C, 40 °C, combined, lyophilised) in mg/100 g. Varieties: JA—Jakubské, BT—Beauty seedless, PE—Perlette, VR—Vrboska, BS—Bezsemenné.

The vitamin C content of dried grapes of different varieties and drying methods is presented in Figure 1. Raisins from the Jakubské variety after lyophilisation had the highest vitamin C content (0.58 mg per 100 g of dried raisins). High values were also found in samples from the Vrboska (0.46 mg/100 g) and Jakubské variety (0.41 mg/100 g) dried at 40 °C and also in lyophilised grapes of the Perlette variety (0.36 mg/100 g). In general, it can be stated that the highest content of vitamin C was found in grapes dried at 40 °C and in lyophilised grapes. The lowest vitamin C content was clearly found in samples dried at 70 °C.

Vitamin C can be considered as one of the factors that determine how gently the food material was processed, because the effects of heat, exposure to oxygen, non-enzymatic changes, and the effects of heavy metals and long periods of storage could reduce nutritional quality [21]. It is known that drying leads to loss of vitamins in dried fruit, particularly of vitamin C content, which is the most sensitive compound to heat, light, and oxygen. This was also confirmed in this study, where increasing temperatures in the drying process led to the reduction of vitamin C content.

Carranza-Concha et al. [22] examined the effect of drying methods on the vitamin C content of dried grapes and came to the same conclusions. Xiao et al. [23] similarly examined the effects of drying temperature on vitamin C retention in dried grapes, then the samples with the highest retention of vitamin C were dried at the lowest drying temperature of 50 °C (2.25 mg/100 g), while vitamin C was broken down at the highest rate at the highest drying temperature of 65 °C (0.57 mg/100 g). Therefore, increasing the drying temperature had a negative effect on vitamin C retention. At the same time, they found that the air speed during drying had no significant impact on vitamin C retention.

A study by Çağlarirmak [21] examined vitamin C levels in sultanas and raisins. It was found that the average vitamin C content in sultanas was 3.67 mg/100 g, which was lower than the average value in raisins (5.15 mg/100 g), which is ten times higher than the amount determined in this study. An even greater amount of vitamin C in dried grapes was established by Clary et al. [24], i.e., 12.5 mg/100 g in grapes dried by microwave vacuum drying and 8.83 mg/100 g in grapes dried in the sun. Thakur et al. [25] examined the impact of processing grapes before drying on the vitamin C content and found that grapes from the Perlette variety dried whole retain the most vitamin C, compared to grapes that were halved or had their skins removed.

Although no significant differences were found between varieties of vitamin C content, increasing drying temperature caused a higher loss of vitamin C. From this point of view, lyophilisation, together with lower temperature, appears to be the best drying method.

### 3.3. Microbiological Analysis

**Figure 2:**
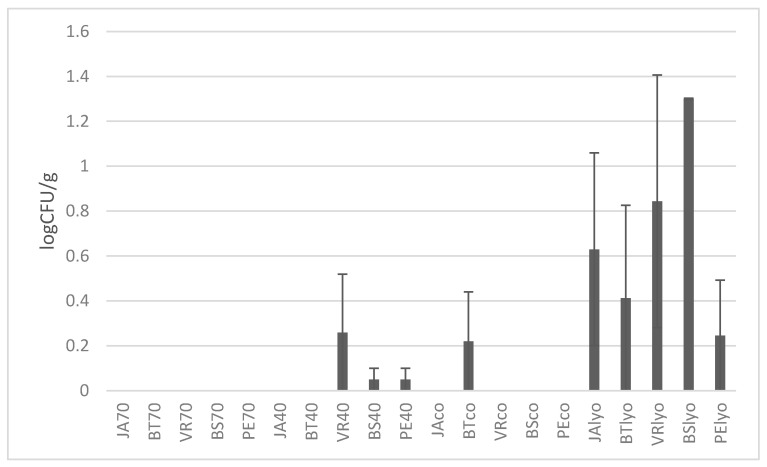
Determination of moulds in individual varieties depending on drying method (70 °C, 40 °C, combined, lyophilised) in log CFU/g. Varieties: JA—Jakubské, BT—Beauty seedless, PE—Perlette, VR—Vrboska, BS—Bezsemenné. CFU—colony forming units.

Figure 2 shows the number of moulds (as log CFU/g; Colony Forming Units) in dried grapes of different varieties and drying methods. The highest value of moulds (CFU/g) was determined in lyophilised grapes of the Bezsemenné variety. Higher amounts of mould were detected in all lyophilised samples, and on the contrary, in samples dried at 70 °C moulds were not detected.

Mould spores can survive in the dried product for a long time, so dried foods cannot be considered sterile. Çağlarirmak [21] found that the development of moulds that produce ochratoxin A depends on the method of drying and the conditions under which the finished product is stored.

Hakobyan et al. [13] analysed 87 samples of dried grapes in which 27 species of moulds from 5 genera were identified: *Mucor, Aspergillus, Penicillium, Trichoderma*, and *Alternaria*, and within them, 13 species (48%) belonged to the genus *Aspergillus*. According to Hakobyan et al. [13], the technological process for drying grapes significantly influences the level of contamination of the final product by fungi and mycotoxins. It was found that the level of contamination of dried fruit is directly dependent on the application of SO_2_. This is an effective preservative and has a significant inhibitory effect on the development of moulds during drying and storage, including the species producing toxins. In this study, the grapes were not treated with any preservative, which can be an explanation for the more extensive development of moulds in lyophilised grapes and dried grapes at 40 °C. Zemni et al. [26] analysed contamination with moulds of Italia Muscat grapes depending on various drying and pre-treatment methods. These authors found that none of the drying or pre-treatment methods prevented the occurrence of moulds completely, which corresponds to this study.

**Figure 3:**
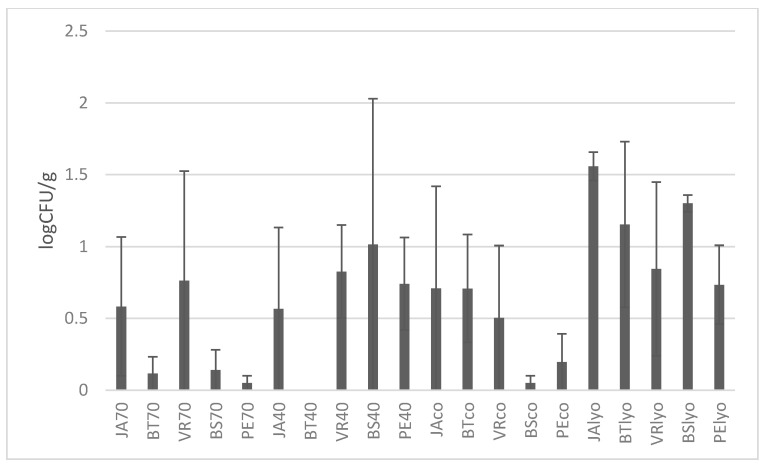
Determination of yeasts in individual varieties depending on drying methods (70 °C, 40 °C, combined, lyophilised) in log CFU/g. Varieties: JA—Jakubské, BT—Beauty seedless, PE—Perlette, VR—Vrboska, BS—Bezsemenné. CFU—colony forming units.

After yeast enumeration, it was established that the samples contained much higher numbers of yeasts than moulds (Figure 3), and lyophilised samples had the highest occurrence. The highest number of yeasts was found in the Bezsemenné variety dried by lyophilisation (1.63 log CFU/g). Samples dried at 70 °C had the best results in microbiology (total count of microorganisms (TCM)) together with the Perlette variety having the lowest number of yeasts (0.1 log CFU/g). No statistically significant difference on the level of significance of *p* ˂ 0.05 was found between the samples.

**Figure 4:**
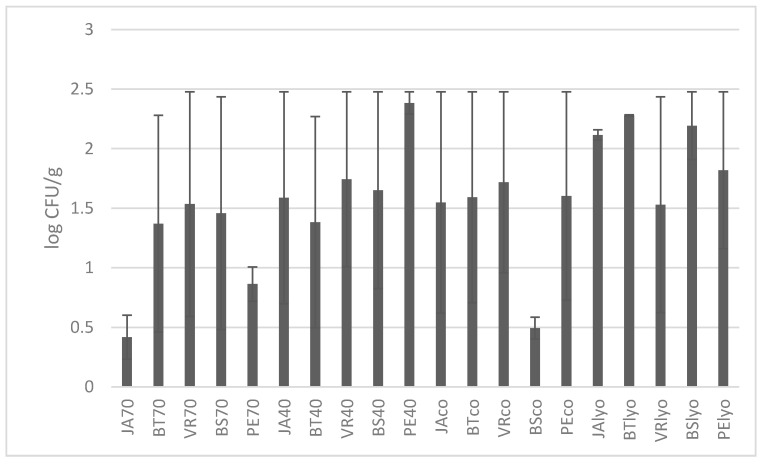
Determination of total count of microorganisms (TCM) in individual varieties depending on drying methods (70 °C, 40 °C, combined, lyophilised) in log CFU/g. Varieties: JA—Jakubské, BT—Beauty seedless, PE—Perlette, VR—Vrboska, BS—Bezsemenné. CFU—colony forming units.

The total number of microorganisms in dried grapes of different varieties and drying methods is shown on Figure 4. The highest value was found in raisins of the Perlette variety dried at 40 °C (2.48 log CFU/g). Generally, higher values were determined in lyophilised grapes, followed by grapes dried at 40 °C. In contrast, the lowest total amount of microorganisms was found in the Jakubské variety dried at 70 °C (0.23 log CFU/g). There were no statistically significant differences between varieties and drying methods within each variety.

It can be concluded that higher temperature during drying had a more detrimental effect on total number of microorganisms, moulds, and yeasts in dried raisins.

McCoy et al. [27] examined the impact of processing on the microbiological properties of unprocessed (obtained from farmers) and processed (obtained from producers) raisins. They came to the conclusion that treatment performed by the producer before processing resulted in raisins with reduced contamination by microorganisms. The processing and level of treatment in the selection of grapes has a major impact on the microbial quality of food. Likewise, various harvesting factors and other factors can cause heterogeneous grape quality, which in our case has resulted in great variability, which in some cases did not give statistically different results.

### 3.4. Instrumental Colour Measuring

Assessment of surface colour for quality assessment is a common practice in dried grape production. Evaluation of dried grape berries is mostly based on visual assessment by trained humans, a process that is subjective. To eliminate errors, it is necessary to combine the results and supplement them with instrumentally based methods with an effort to objectify the results, such as colour measurement [28]. Colour measuring via CIE L*a*b* values is the way of rapid and objective quality evaluation of grape quality and is a usable method for replacing subjective fruit grading by humans. The colour of the dried grapes is subsequently darker with lower L* and with changed colour coordinates a* and b* than the colour of fresh grapes. This depends more on the skin colour of grapes given by variety as was found by Adiletta et al. and Serratosa et al. [10,29]. In Table 3, there are data with expressed differences (*p* ˂ 0.05) depending on the colour parameter.

The white grape varieties, Perlette and Bezsemenné, had increased L* and b* values especially after lyophilisation. This was not the case with the pink variety (Beauty seedless). The colour change ΔE*_ab_ was more pronounced in white grapes varieties. The lowest total colour change (ΔE*_ab_ = 2.24) was in the Jakubské grape variety, while the other blue variety Vrboska had very prominent colour difference after lyophilisation. It can be concluded that white grape varieties were characterised by higher colour change after drying, than the blue or pink grape varieties.

Just like sensory analysis, instrumental colour may not be essential for expressing differences depending on the processing method and expression for overall quality. The overall quality of food must be co-created by comprehensive results, and it is therefore desirable to create an appropriate combination of available methods for food evaluation [2,30]. It is important to note that any change in appearance from the standard may be essential for the consumer [31]. It also depends on who evaluates the colour of the product, i.e., the consumer or the producer. Measuring colours as a quality parameter in the consumer’s choice and purchase of food may not be as important to overall quality. On the other hand, for the drying process, the colour is an element of controlling and standardising the final product.

### 3.5. Sensory Atributes

The appearance, texture, taste, and odour, along with the nutritional value, are properties that have the greatest impact on the consumer when choosing a food product. The results of sensory analysis are shown in Table 4.

In general, we can state that grapes dried using lyophilisation had the best results for evaluation of the smoothness of the surface. There is growing interest in drying using lyophilisation in the food industry, because these products have noticeable quality compared to other methods of drying. Lyophilisation takes place at low temperatures, which maintains flavour, colour, and appearance. This also minimises thermal damage to heat-sensitive nutrients. Because the entire process takes place in a solid state, shrinkage and other changes to structure are reduced substantially [32]. This fact was confirmed during sensory evaluation. Lyophilised raisins did not have a wrinkled surface, as contrary to the samples of the Jakubské variety that were dried at 70 °C and evaluated as the most wrinkled. On the other hand, the highest values of gloss were established in lyophilised samples and in samples dried at 70 °C.

According to many studies, a linear relationship between wrinkling, the degree of moisture, and temperature has been observed during drying. The reason for this is the fact that drying leads to changes to the food on a micro-structural level, and this subsequently influences its macroscopic properties. Loss of water and segregation of components cause damage and disruption to cellular walls and even disintegration of cellular tissue. In general, these changes are linked to a reduction of the volume of the product [2]. Angulo et al. [33] found that the type of cultivar and also the method of drying influence the degree of wrinkling.

The colour of dried grapes was generally rated positively, lyophilised grapes were again rated with higher values. The Jakubské variety was also highly rated when dried by combined methods and at a temperature of 40 °C. Within most methods of drying, a downward tendency was noticed from samples of the Jakubské variety through Beauty seedless, Vrboska, and Bezsemenné to Perlette, which scored smaller values. When evaluating the colour, no statistically significant difference was found at a level of significance of *p* < 0.05. The brown colour of dried grapes originates from the effects of enzymatic reactions (the effects of the polyphenol oxidase enzyme) and non-enzymatic browning (Maillard reaction). The colour of dried raisins is subsequently darker than the colour of fresh grapes [10,29]. Chervin et al. [34] stated that the sugar content of the grapes also influences how brown they become during drying, as well as the method of drying. Simal et al. [8] noticed, interestingly, that two different preferences became apparent among the assessors: one group of assessors preferred lighter grapes, while the other group preferred darker grapes. In our study, the assessors were also divided into two groups by preference, into a group that preferred darker grapes, i.e., the most similar to classic raisins, and the other group, which preferred lyophilised grapes, i.e., with a colour most similar to fresh grapes.

When evaluating odour, it is not clear which method of drying or which variety was better, because the results were very similar. According to the assessors, the lyophilised grapes of the Jakubské variety had the most intense aroma (79.9). The grapes of the Bezsemenné variety dried at 40 °C were rated as the second most intense odour (75.8). No statistically significant differences between the samples were found. According to literature, there are three ways that aromatic substances can originate in the dried grapes. Most of them come from the fresh grapes, other metabolites such as furans and pyrazines are formed during drying as a result of the Maillard reactions, and the third is origination of aromatic substances (aliphatic compounds such as acids and aldehydes) by oxidation of unsaturated fatty acids [2].

Lyophilised grapes of the Jakubské variety had the highest chewability followed by grapes of the Beauty seedless variety dried at 40 °C. Raisins from the Vrboska variety dried at 70 °C were rated with smaller values. Grapes dried at 70 °C were generally rated as difficult to chew and tough. Xiao et al. [23] examined the impact of various drying temperatures (50, 55, 60, and 65 °C) on the texture and hardness of grapes from the Monukka seedless variety. Drying temperatures ranging from 55 to 65 °C increased the hardness of grapes significantly. This finding was explained by the fact that high temperature caused water to evaporate from the surface of the grapes and poor migration of water from inside the fruit, which led to formation of a hard surface layer. According to Angulo et al. [33], the cultivar type and the drying method influenced the majority of the textural properties of the grapes. The raisins that were the stickiest or chewiest generally contained the least water.

The juiciness of the grapes was generally rated uniformly, but the Beauty seedless variety dried at 40 °C had the highest value; in contrast, grapes that were nearly totally dry and sticky were grapes from the Vrboska variety dried at 70 °C.

According to the assessors, the Bezsemenné variety dried at 40 °C had the most intense taste, followed by lyophilised grapes of the same variety. Grapes of the Jakubské variety dried at 70 °C had the lowest score, meaning the most unpleasant and atypical taste. No statistically significant differences in taste between drying treatment within varieties were found. Taste and odour are considered important attributes of quality, which, along with appearance, influence the saleability of the product. While volatile components influence both taste and odour, non-volatile compounds only influence taste. Odour and taste are probably the parameters that are the most difficult to determine because they are very subjective. Their evaluation mostly depends on the consumer’s personal preferences as stated by Khiari et al. [2].

Angulo et al. [33] examined two cultivars and two methods of drying raisins and found that all raisins had the same caramel flavour, but all other attributes differed depending on the kind of cultivar, the drying method, or both. The grapes differed in sweetness, acidity, and astringency depending on the drying method. Gee [35] examined the impact of processing grapes on the flavour properties. Mechanically dried, chopped raisins had a rich taste reminiscent of the concentrated fresh grape. The taste of whole brown raisins was similar to the typical taste of raisins.

At the end, the assessors were also required to record the overall impression in a sensory questionnaire. Generally, it was established that overall impression values correspond with the taste values. The Beauty seedless variety dried at 40 °C had the best overall impression, immediately followed by the Bezsemenné variety prepared by lyophilisation. The Jakubské variety dried at 70 °C scored the smallest values. No statistically significant differences in overall impression were found between drying treatment within varieties.

In general, it can be stated that panellists rated samples of raisins dried at 40 °C and lyophilised samples with higher scores. Samples of grapes dried at the highest temperature, 70 °C, were rated with smaller values. Angulo et al. [33] found that raisins produced from the fruit of various varieties of grape and using various drying methods had unique sensory properties that influenced consumer preferences: whereas some consumers preferred a specific variety regardless of drying method, others preferred a specific drying method regardless of variety.

### 3.6. Principal Component Analysis

**Figure 5:**
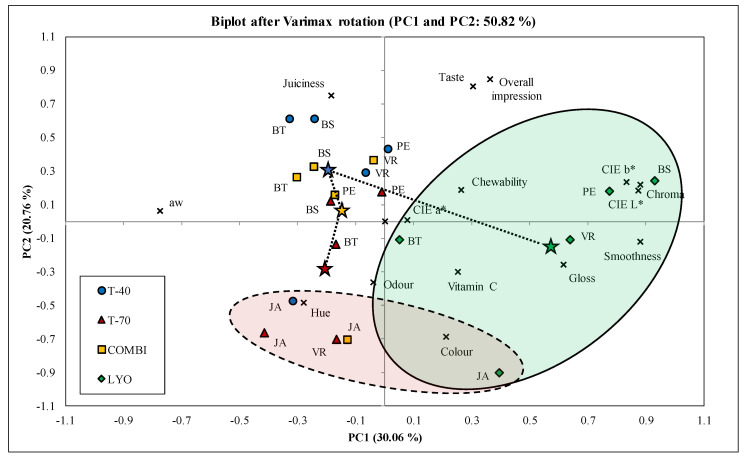
Principal component analysis biplot for the analysed attributes (marked with “X”) and samples (JA—Jakubské, BT—Beauty seedless, PE—Perlette, VR—Vrboska, BS—Bezsemenné) considering drying methods (T-40—40 °C, T-70—70 °C, COMBI—combined, LYO—lyophilised).

The principal component analysis (PCA) was conducted with the aim of determining the most suitable variables for elaboration and description of dried raisins. The first two principal components (PCs) accounted for 50.82% of variance (PC1 = 30.06%, PC2 = 20.76%; Figure 5). The relations between variables and PCs were interpreted according to the correlations between them. Thus, variables that were close to each other were considered positively correlated and those separated by 180° as negatively or by 90° as independently correlated. PC1 was positively correlated with the colour variables (CIE L*, b*, and chroma) and smoothness, and negatively with a_w_ value. PC2 was mostly defined by sensory variables, although this link was weaker because of smaller squared cosines. Nevertheless, PC2 was correlated with overall impression, taste, and juiciness on the positive side, and with colour on the negative side. It was found that sensory traits such as chewability, odour, and gloss on one hand, and the content of vitamin C on the other had low impact on raisin characterisation. As expected, overall impression was close to taste, meaning high correlation. Although those traits had high contribution to PC2 definition, none of the observations were close to them. Therefore, it can be concluded that the taste and overall impression were not key determinants of any variety or drying treatment.

There was no clear separation of drying treatments and grape varieties, and a lot of overlapping was observed (Figure 5). However, three observations could be elaborated. The first observation is related to the lyophilisation treatment of all grape varieties, which was placed in the right quadrant (green ellipse) where colour traits and appearance were placed. This could lead to conclusion that among all examined drying treatments lyophilisation had the highest impact on preserving desirable visual attributes. Other drying treatments did not have such a distinctive spatial distribution and were mixed. The second observation is related to the change of sensory attributes with drying treatments marked with connected stars. As stated, colour and appearance traits were related to drying treatment at lower temperature (lyophilisation) labelled with green star. With increasing drying temperature, there is a transition from visual traits to juiciness, which was related mostly with drying at lower temperatures at 40 °C (blue star). With further increase in temperature during combi treatment (yellow star) and especially at 70 °C (red star), the relation to juiciness decreased, and link to hue and desirable odour increased. Third observation is related to the Jakubské grape variety (red ellipse), which was placed closely in lower quadrants where colour, hue, and odour had high loadings. It appears that this variety has pronounced characteristics that dominate over different drying treatments giving a uniform final product. Additionally, this variety was placed opposite to the taste and overall impression, leading to low likeability of that variety. After checking the analysed parameters, we can infer the essential role played by the used grape varieties in drying processing, and as a result, in obtaining the final product, regardless of whether they are grapes or wine products [36].

## 4. Conclusions

This study examined the impact of drying methods on the quality of five varieties of grape dried at different temperatures or lyophilised on the bunch stem, it could affect processing companies. We can state that sensory traits of lyophilised raisins were rated with higher values, followed by grapes dried at 40 °C. Raisins dried at the highest temperature, this being 70 °C, scored smaller values. Similar results were achieved when evaluating vitamin C content, where the highest content was recorded in lyophilised grapes and in grapes dried at 40 °C, while the lowest content was found in grapes dried at 70 °C. No significant differences between varieties in vitamin C content were found. With regard to microbiological analysis, the results showed the opposite trend to sensory evaluation and vitamin C content, which means that the lowest level of contamination by microorganisms was recorded in grapes dried at 70 °C, which was expected with regard to the fact that the grapes were not treated with any preservative. Nevertheless, lyophilisation as a gentle method for vitamin C content affected microbial quality, especially count of moulds and yeasts. PCA analysis revealed that colour traits, smoothness, juiciness, taste, and overall impression were the most important variables for principal components definition. Although observations were not clearly separated, several conclusions could be stated, which corresponds to the discussion on the analysis performed. A pattern of attribute change with increase in temperature was perceived, from visual appreciation at lyophilisation temperature, through juiciness at mild temperatures, to odour and colour appreciation at higher temperatures. All selected varieties have achieved adequate quality parameters. Varieties Vrboska and Bezsemenné appear to be the positive rated.

## Figures and Tables

**Table 1:** Description of grape varieties included in the experiment. VIVC—Vitis International Variety Catalogue.

Variety	Designation	Grapes Colour	Grapes Description	Origin/Variety Number VIVC
Jakubské	JA	blue	medium large, cylindrical shape, seeds	France/9280
Beauty seedless	BT	blue	large, egg-shaped, seedless	USA/1065
Vrboska	VR	pink	medium large, conical shape, seeds	Czech Republic/17515
Bezsemenné	BS	white	large, round shape, seedless	Czech Republic/new cultivar AMPELOS
Perlette	PE	white	large, cylindrical shape, seedless	USA/9168

**Table 2:** Determining pH and total soluble solids (TSS) content of fresh grapes.

Variety	pH	Total Soluble Solids (°Brix)
JA	3.21 ± 0.03 ^b^	13.50 ± 0.29 ^ab^
BT	3.36 ± 0.01 ^a^	14.33 ± 0.66 ^ab^
VR	3.37 ± 0.01 ^a^	15.67 ± 0.66 ^b^
BS	3.14 ± 0.01 ^b^	15.00 ± 0.60 ^ab^
PE	3.42 ± 0.01 ^a^	13.33 ± 1.45 ^a^

Notes: The values marked by different indexes (^a^, ^b^) in the same column differ significantly at *p* < 0.05. Varieties: JA—Jakubské, BT—Beauty seedless, PE—Perlette, VR—Vrboska, BS—Bezsemenné.

**Table 3:** Colour determination of fresh and dried grape varieties depending on drying method (fresh—none, 70—70 °C, 40—40 °C, co—combined, lyo—lyophilised).

Variety	Parameter	Fresh	40	70	co	Lyo
PE	L*(D65)	48.71 ± 3.85 ^a^	27.40 ± 2.46 ^b^	27.47 ± 1.83 ^b^	26.42 ± 1.31 ^b^	67.37 ± 3.09 ^c^
	a*(D65)	−2.92 ± 1.12 ^a^	5.88 ± 0.88 ^b^	3.74 ± 0.62 ^c^	5.59 ± 1.56 ^b^	−2.64 ± 1.35 ^a^
	b*(D65)	20.23 ± 3.20 ^a^	7.14 ± 1.53 ^b^	4.22 ± 0.59 ^c^	5.60 ± 1.83 ^bc^	42.62 ± 3.10 ^d^
	ΔE*_ab_	0.00	26.51 ***	27.42 ***	27.99 ***	29.15 ***
BS	L*(D65)	39.70 ± 1.31 ^a^	28.34 ± 0.86 ^b^	19.96 ± 1.32 ^c^	26.56 ± 0.62 ^b^	71.88 ± 3.87 ^d^
	a*(D65)	−3.39 ± 0.64 ^a^	3.09 ± 0.57 ^b^	2.65 ± 0.54 ^b^	3.17 ± 0.42 ^b^	−5.04 ± 3.64 ^a^
	b*(D65)	12.16 ± 2.17^a^	2.58 ± 0.74 ^b^	2.58 ± 0.46 ^b^	4.61 ± 0.66 ^c^	42.34 ± 3.65 ^d^
	ΔE*_ab_	0.00	16.21 ***	22.76 ***	16.51 ***	44.15 ***
JA	L*(D65)	27.96 ± 1.50 ^a^	24.81 ± 2.79 ^b^	22.05 ± 1.30 ^c^	25.24 ± 1.27 ^b^	29.80 ± 2.44 ^a^
	a*(D65)	0.40 ± 0.31 ^a^	0.64 ± 0.44 ^a^	2.10 ± 0.58 ^b^	0.97 ± 0.48 ^a^	1.50 ± 2.53 ^ab^
	b*(D65)	−1.47 ± 0.69 ^a^	−1.94 ± 0.84 ^a^	−0.35 ± 0.25 ^b^	−0.57 ± 0.30 ^b^	−0.86 ± 0.91 ^ab^
	ΔE*_ab_	0	3.19 *	6.25 **	2.91	2.24
VR	L*(D65)	28.85 ± 0.81 ^a^	25.30 ± 1.64 ^b^	25.02 ± 1.65 ^b^	29.14 ± 1.92 ^a^	41.17 ± 6.22 ^c^
	a*(D65)	8.59 ± 1.30 ^a^	4.36 ± 0.80 ^b^	3.31 ± 0.61 ^b^	4.82 ± 0.44 ^b^	23.39 ± 4.74 ^c^
	b*(D65)	3.62 ± 0.96 ^a^	2.98 ± 1.37 ^a^	2.51 ± 1.04 ^a^	5.61 ± 0.91 ^b^	13.04 ± 3.40 ^c^
	ΔE*_ab_	0	5.56 *	6.61 **	4.28 *	21.43 ***
BT	L*(D65)	28.67 ± 2.10 ^a^	27.32 ± 0.89 ^ac^	23.42 ± 1.46 ^b^	26.10 ± 1.31 ^c^	22.90 ± 1.95 ^b^
	a*(D65)	0.65 ± 0.40 ^a^	−0.02 ± 0.19 ^b^	0.67 ± 0.19 ^a^	1.38 ± 0.63 ^c^	−0.01 ± 0.37 ^b^
	b*(D65)	−1.99 ± 0.93 ^a^	−3.07 ± 0.51 ^b^	−0.49 ± 0.11 ^c^	−0.50 ± 0.24 ^c^	−2.22 ± 0.76 ^a^
	ΔE*_ab_	0	1.84	5.45 *	3.05 *	5.81 *

Notes: Results are expressed as mean value ± standard deviation. Different letters in superscript indicate significant differences (*p* < 0.05) between the samples in the same row. Designation * means medium colour difference (ΔE*_ab_ = 3.0−6.0), ** prominent colour difference (ΔE*_ab_ = 6.0−12.0), *** very prominent colour difference (ΔE*_ab_ > 12.0). Varieties: JA—Jakubské, BT—Beauty seedless, PE—Perlette, VR—Vrboska, BS—Bezsemenné.

**Table 4:** Sensory evaluation of varieties depending on drying method (70—70 °C, 40—40 °C, co—combined, lyo—lyophilised).

Variety		Smoothness	Gloss	Colour	Odour	Chewability	Juiciness	Taste	Overall Impression
JA	70	11.3 ± 3.3 ^a^	10.0 ± 2.0 ^a^	65.6 ± 8.8	74.3 ± 6.9	57.4 ± 9.0 ^a^	43.0 ± 7.3	52.7 ± 9.8	51.1 ± 7.9
	40	22.0 ± 4.0 ^a^	12.9 ± 2.6 ^a^	76.3 ± 5.4	71.9 ± 6.2	66.6 ± 8.0 ^ab^	59.0 ± 7.2	64.6 ± 8.5	55.8 ± 7.9
	co	17.8 ± 5.1 ^a^	26.5 ± 7.7 ^b^	78.4 ± 4.9	71.9 ± 7.3	68.3 ± 9.4 ^ab^	44.0 ± 8.3	64.3 ± 9.6	53.5 ± 9.5
	lyo	72.9 ± 5.8 ^b^	58.3 ± 6.8 ^c^	78.3 ± 8.2	79.9 ± 4.8	78.2 ± 7.2 ^b^	42.6 ± 10.1	67.9 ± 8.4	63.4 ± 8.6
PE	70	27.2 ± 3.6 ^a^	44.7 ± 8.2	54.5 ± 10.6	62.6 ± 8.4	61.5 ± 6.8 ^ab^	57.1 ± 4.4	73.0 ± 5.2	71.5 ± 5.4
	40	25.5 ± 5.6 ^a^	34.0 ± 7.1	49.9 ± 10.0	69.8 ± 4.8	71.0 ± 5.9 ^b^	67.2 ± 6.7	74.9 ± 6.9	73.3 ± 6.4
	co	21.8 ± 5.6 ^a^	22.6 ± 6.3	49.5 ± 9.9	67.4 ± 4.5	58.6 ± 5.5 ^a^	54.3 ± 5.4	65.7 ± 6.2	64.2 ± 5.8
	lyo	46.1 ± 4.1 ^b^	43.1 ± 7.6	55.8 ± 10.1	61.1 ± 7.0	60.5 ± 7.0 ^ab^	45.0 ± 7.1	74.3 ± 6.3	73.0 ± 6.6
BS	70	22.1 ± 5.8 ^a^	15.7 ± 6.3	58.2 ± 8.4	72.0 ± 7.0	40.7 ± 6.9 ^a^	47.8 ± 7.9	79.0 ± 4.5	66.8 ± 7.5
	40	24.7 ± 3.7 ^a^	14.0 ± 4.8	52.9 ± 9.1	75.8 ± 5.8	66.0 ± 9.0 ^ab^	65.5 ± 7.8	86.0 ± 2.9	75.8 ± 5.1
	co	13.4 ± 3.3 ^a^	15.4 ± 4.3	55.5 ± 8.3	63.6 ± 5.8	56.4 ± 7.4 ^ab^	50.3 ± 8.9	76.6 ± 5.6	69.0 ± 6.1
	lyo	70.3 ± 4.4 ^b^	26.9 ± 9.4	69.5 ± 9.9	73.8 ± 8.7	70.7 ± 8.9 ^b^	56.1 ± 8.8	82.2 ± 6.6	78.6 ± 6.3
VR	70	20.8 ± 4.4 ^a^	24.5 ± 6.3	62.7 ± 7.3	72.4 ± 5.8	33.0 ± 7.0 ^a^	28.3 ± 5.0 ^a^	58.4 ± 8.4	51.4 ± 4.3
	40	35.4 ± 4.9 ^a^	20.9 ± 5.2	62.9 ± 7.9	72.7 ± 5.1	65.0 ± 6.9 ^b^	66.1 ± 6.7 ^c^	80.8 ± 3.7	72.7 ± 4.3
	co	28.7 ± 5.2 ^a^	21.1 ± 5.8	59.4 ± 9.2	67.4 ± 4.2	66.0 ± 6.3 ^b^	61.2 ± 5.1 ^c^	80.9 ± 4.4	71.9 ± 5.2
	lyo	59.0 ± 4.4^b^	48.2 ± 8.6	73.8 ± 8.2	62.8 ± 5.6	63.3 ± 7.2 ^b^	45.1 ± 8.3 ^b^	79.5 ± 6.6	70.3 ± 7.1
BT	70	16.3 ± 6.1 ^a^	46.8 ± 9.2 ^a^	67.2 ± 6.2	61.8 ± 5.9	48.9 ± 8.0 ^a^	44.8 ± 8.7	73.0 ± 5.9	68.4 ± 6.8
	40	19.1 ± 5.8 ^a^	20.9 ± 5.2 ^b^	62.0 ± 6.1	68.0 ± 5.7	74.2 ± 5.1 ^b^	76.4 ± 4.9	80.6 ± 6.2	81.4 ± 5.8
	co	19.4 ± 5.5 ^a^	19.1 ± 5.1 ^b^	63.4 ± 5.6	65.8 ± 3.8	66.3 ± 6.4 ^b^	58.6 ± 5.3	75.6 ± 6.1	72.5 ± 6.3
	lyo	46.0 ± 5.9 ^b^	37.4 ± 5.2 ^a^	73.7 ± 5.4	69.9 ± 0.6	66.1 ± 7.4 ^b^	53.8 ± 7.4	75.6 ± 6.2	73.7 ± 7.9

Notes: Values identified by different indexes (^a^, ^b^, ^c^) in the same column within variety differ significantly at *p* < 0.05. Varieties: JA—Jakubské, BT—Beauty seedless, PE—Perlette, VR—Vrboska, BS—Bezsemenné.
